# Gut Microbiota Shapes the Efficiency of Cancer Therapy

**DOI:** 10.3389/fmicb.2019.01050

**Published:** 2019-06-25

**Authors:** Weidong Ma, Qixing Mao, Wenjie Xia, Gaochao Dong, Changhua Yu, Feng Jiang

**Affiliations:** ^1^Department of Thoracic Surgery, Jiangsu Cancer Hospital, Jiangsu Institute of Cancer Research, Nanjing Medical University Affiliated Cancer Hospital, Nanjing, China; ^2^The Fourth Clinical College of Nanjing Medical University, Nanjing, China; ^3^Department of Radiotherapy, Huai’an First People’s Hospital, Nanjing Medical University, Huai’an, China; ^4^Jiangsu Key Laboratory of Molecular and Translational Cancer Research, Nanjing Medical University Affiliated Cancer Hospital, Cancer Institute of Jiangsu Province, Nanjing, China

**Keywords:** gut microbiome, cancer therapy, immunotherapy, chemotherapy, radiotherapy

## Abstract

Systems biology provides an opportunity to discover the role that gut microbiota play in almost all aspects of human health. Existing evidence supports the hypothesis that gut microbiota is closely related to the pharmacological effects of chemical therapy and novel targeted immunotherapy. Gut microbiota shapes the efficiency of drugs through several key mechanisms: metabolism, immunomodulation, translocation, enzymatic degradation, reduction of diversity, and ecological variability. Therefore, gut microbiota have emerged as a novel target to enhance the efficacy and reduce the toxicity and adverse effects of cancer therapy. There is growing evidence to show that cancer therapy perturbs the host immune response and results in dysbiosis of the immune system, which then influences the efficiency of the therapy. Studies suggest that gut microbes play a significant role in cancer therapy by modulating drug efficacy, abolishing the anticancer effect, and mediating toxicity. In this review, we outline the role of gut microbiota in modulating cancer therapy and the implications for improving the efficacy of chemotherapy and immunotherapy in clinical practice. We also summarize the current limitations of the safety and effectiveness of probiotics in cancer therapies such as personalized cancer therapy.

## Introduction

Microbial communities have evolved into a diverse array of specialized lineages in order to adapt to different habitats and which have shaped the evolution of modern life (Dzutsev et al., 2017). The development of next generation sequencing has made the identification and relative quantitation of species more precise than can be acheieved by traditional methods. Thus, microbiotic responses to microenvironmental changes are being elucidated.

Gut microbes have a role in shaping normal and pathologic immune responses to cancer therapy. A host’s mucosal immune system and microbial communities are coevolutionary, and multiple mechanisms have been developed for maintaining homeostasis. However, when pathogenic bacteria disturb this tightly balanced ecosystem, the immune system responds to the bacteria and may also change the immune response to tumors and the tumor microenvironment (Gagliani et al., 2014). As surgical treatment and chemotherapy and radiotherapy regimens become increasingly efficacious, cancer survival rates have dramatically improved in recent decades (Siegel et al., 2018). For most patients with advanced disease, cytotoxic drugs are the mainstay of medical treatment. However, these drugs can cause considerable treatment-related morbidity and mortality and unpredictable treatment response. Idiosyncratic adverse effects, acquired resistance, and high costs are issues for targeted therapies. Intestinal microbiota can provide a novel way to enhance the efficacy and reduce the toxicity of current chemotherapeutic drugs and improve sensitivity to immunotherapy.

The host’s diet feeds and shapes the microbiome to satisfy the nutritional needs of the host (Dzutsev et al., 2017). In the metaorganism, crosstalk between host and commensal microbes is beneficial to the maintenance of physiological homeostasis (Dzutsev et al., 2017). It is widely accepted that microbiota at the epithelial barrier affect host systemic functions such as nutrition, metabolism, energy balance, inflammation, and adaptive immunity (Cryan and Dinan, 2012; Zeevi et al., 2015). The microbiota do not usually elicit a proinflammatory immune response because the host mucosal immune system coevolves with commensal organisms. Hosts have developed multiple mechanisms to maintain ecological balance.

When pathogenic bacteria disturb this balanced ecosystem and impair these mechanisms, the responses of the immune system to the microbiota may also change the immune response. Gut microbes can, therefore, shape normal and pathologic immune responses to cancer therapy. Recent human clinical studies, meta-analyses of clinical studies, and preclinical studies using cell culture and animal models have revealed that gut microbiota play various roles in the host response to different anticancer drugs. One of the central mechanisms may be immunomodulation. Dysbiosis may be both the result of tumor therapy and the cause of differential responses to tumor therapy (Bhatt et al., 2017). Here, we outline how gut bacteria influence the effects of chemotherapy and immunotherapy (Table 1).

**Table 1:** Summary of the effects of gut microbiota on tumor treatment.

Therapy	Side effect	Relevant mechanism
Irinotecan	Diarrhea	Microbiota can reactivate SN38 by secreting b-glucuronidase enzymes (Lin et al., 2012)
Doxorubicin	Intestinal mucositis	Significant changes in microbiota occur in the oral cavity and the intestinal tract (Napeñas et al., 2010; Rigby et al., 2016)
5-FU	Intestinal dysbiosis	*Staphylococcus* and *Clostridium* species increase and *Bacteroides* and *Lactobacillus* decrease (Stringer et al., 2009b)
Ionizing radiation therapy	Oral mucositis, enteritis, colitis, diarrhea and bone marrow failure	RTX alters the microbiota composition, breaks the intestinal barrier and causes apoptosis in intestinal crypts (Barker et al., 2015)
Total body irradiation	Radiotherapy toxicity	The expression of ANGPTL4 is restrained by the gut microbiota to induce apoptosis in endothelial cells of the intestinal mucosa (Crawford and Gordon, 2005)


## Gut Microbiota and the Efficiency of Cancer Therapies (Including Chemotherapy, Radiotherapy, and Immunotherapy)

### Immunotherapy

Immunotherapy has been very successful in the treatment of cancer. Identification and killing of tumor cells partly depends on T cell-mediated cellular immunity. T cells, through T cell receptors (TCR), combine with a specific antigen of the major histocompatibility complex (MHC) on the surface of tumor cells. The interaction of TCR and MHC molecules is controlled by a series of immune checkpoints, with costimulatory signals and coinhibitory signals that can activate or inhibit T cells. Cytotoxic T lymphocyte-associated protein 4 (CTLA-4), programmed cell death 1 (PD-1), and PD-1 ligand (PD-L1) are coinhibitory molecules that can restrain the immune response to prevent autoimmune diseases. In the tumor microenvironment, stromal cells and cancer cells often overexpress coinhibitory ligands and receptors. PD1 and its ligand PD-L1 play important roles in immune tolerance. Their binding can transmit coinhibitory signals that inhibit the immune activity of T cells and can cause immune escape of tumor cells (Sharma and Allison, 2015). To date, CTLA-4 and the PD-1/PD-L1 axis (mAb-mediated blockade of two checkpoints) have produced the greatest clinical success (Sharma and Allison, 2015). Monoclonal antibodies against PD-1 (nivolumab), PD-L1 (pembrolizumab), and CTLA-4 (ipilimumab) have already received FDA approval for several cancers. These monoclonal antibodies can reactivate the patient’s own immune response against tumors. These antibodies have been highly effective for treating Hodgkin lymphoma, melanomas, kidney cancer, lung cancer, and bladder cancer. Although these findings are promising, patients’ responses to checkpoint inhibitors have considerable inter-individual variation, as seen with other cancer therapies (Vétizou et al., 2015; Pitt et al., 2016a,b). The cause of this heterogeneity in response remains unclear, however, and elucidating the cause could boost the efficacy of treatment and expand the respondent population. Interestingly, recent human clinical studies and preclinical trials have suggested that the efficacy of checkpoint inhibitors is affected by patients’ gut microbiota. The observed variation in clinical responses may be explained by the interaction between the gut microbiota and immune checkpoint inhibitors (Sivan et al., 2015; Vétizou et al., 2015).

Sivan et al. (2015) used mouse models of melanoma and found that gut microbiota accounted for the variation in clinical responses to immune checkpoint inhibitors. They noted that different laboratory mice had different tumor growth speeds and that tumors grew more slowly and responded more effectively to anti-PD-L1 in Jackson Laboratory (JAX) mice than in Taconic mice. These mice had the same genetic background, but their microbial compositions were distinct. When JAX donors’ fecal microbiota was transplanted into Taconic recipients, anti-PD-L1 antitumor efficacy was enhanced. *Bifidobacterium* was identified as being crucial, and feeding *Bifidobacterium* alone could enhance anti-PD-L1 efficacy by reactivating dendritic cells that boosted CD8-positive T cell responses to defeat tumors (Sivan et al., 2015).

Zitvogel et al. (Routy et al., 2018) revealed an interesting phenomenon in which antibiotics made patients relapse sooner and shortened their survival. Patients who did not receive antibiotics before, or soon after, anti-PD1 had a better response to anti-PD-L1 (Routy et al., 2018). Based on an analysis of the microbiota composition of 100 lung and renal cancer patients treated with anti-PD1 gut microbiota in both Europe and the United States, the bacterial species *Akkermansia muciniphila* was shown to be significantly more abundant in anti-PD1 responders (R) than non-responders (NR) (Routy et al., 2018). To determine whether gut microbiota play a key role in the different responses to anti-PD1, the researchers transplanted the patients’ fecal microbiota into antibiotic-treated mice or germ-free mice and noted that these mice acquired the same ability to respond to immune checkpoint blockade (ICB). The studies also showed a higher frequency of *Enterococcus hirae* in R patients and a trend of higher representation of *Corynebacterium aurimucosum* and *Staphylococcus haemolyticus* in NR patients (Routy et al., 2018). NR patients’ fecal microbiome could not replicate the mouse response to anti-PD1, but the unresponsiveness could be rescued by gavage with *A. muciniphila* alone or in combination with *E. hirae* (Routy et al., 2018). *A. muciniphila* can cause IL-12 production and increase gut-tropic CD4+ T cells, which express the chemokine receptor CCR9 in tumor beds, tumor-draining lymph nodes, and mesenteric lymph nodes to exert an adjuvant effect on the anti-PD1 response. *A. muciniphila* is an elliptic gram-negative bacterium that preferentially colonizes the mucus layer of the gut. Studies have shown that metformin improves the abundance of intestinal *A. muciniphila* (Lee et al., 2017). These findings suggest that metformin could be used to increase the sensitivity of tumor patients to immune checkpoint inhibitors. Additional research is needed to confirm this finding.

Wargo et al. (Gopalakrishnan et al., 2018) at the MD Anderson Cancer Center explored *Faecalibacterium* species enriched in R patients by 16S rRNA gene sequencing in 25 samples from melanoma patients treated with anti-PD1 (Vétizou et al., 2015; Gopalakrishnan et al., 2018). *Faecalibacterium* showed a significant positive correlation with progression-free survival, while *Bacteroidales* increased the risk of relapse. Patients with a higher abundance of *Faecalibacterium* at the treatment baseline had preexisting anticancer immune responses, and a higher number of cytotoxic CD8 + T cells were found to have infiltrated the tumor bed. This result could be replicated in a mouse model. In a similar case, Gajewskis et al. (Matson et al., 2018) (University of Chicago, IL, United States) analyzed 38 fecal samples from metastatic melanoma patients undergoing anti-PD1 treatment and identified that *Bifidobacterium longum*, *Enterococcus faecium*, and *Collinsella aerofaciens* contributed to a better prognosis. Germ-free mice with an R patient fecal microbiota transfer had better tumor control and responded more strongly to anti-PD-L1 (Matson et al., 2018).

**FIGURE 1:**
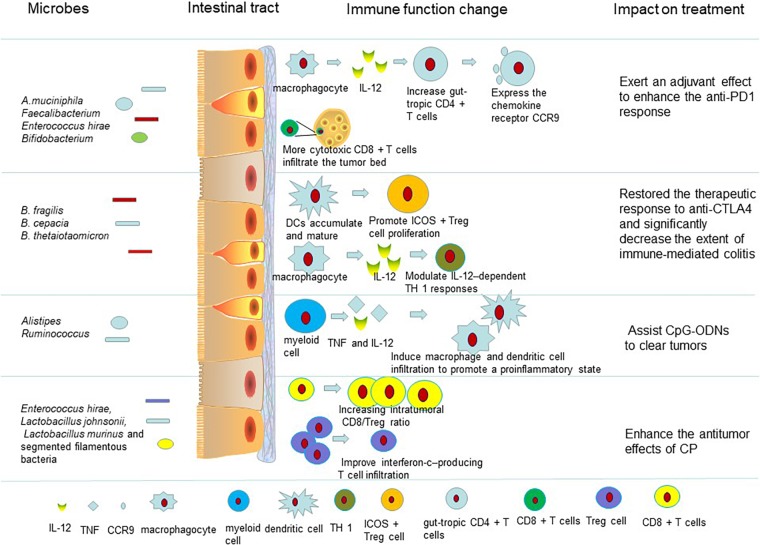
Intestinal microbiota influence the therapeutic effect of drugs on tumors by regulating the immune system. The gut microbiota enhance anti-PD-L1 efficacy by reactivating dendritic cells. Dendritic cells can boost CD8-positive T cell responses and increase the number of gut-tropic CD4 + T cells to defeat tumors. Also the gut microbiota can trigger dendritic cell maturation and modulate IL-12–dependent TH 1 responses to restore the therapeutic response to anti-CTLA4. The microbiota are associated with side effects of immunotherapy (Supplementary Table S1). When germ-free mice receiving anti-CTLA-4 mAb were monopolized with *B. fragilis*, plasmacytoid DCs accumulated and matured in mesenteric lymph nodes. Dendritic cells can promote ICOS + Treg cells to proliferate in the lamina propria. The gut microbiota help CpG-ODNs to promote myeloid cells to secrete proinflammatory cytokines such as TNF and IL-12. TNF and IL-12 induce macrophage and dendritic cell infiltration to promote a proinflammatory state (Supplementary Table S1). The body develops an antigen-specific adaptive T cell antitumor immunity to clear tumors in this proinflammatory microenvironment. Some of the intestinal microbiota affect the antitumor efficacy of CP. *E. hirae* translocation could improve the intratumoral CD8/Treg ratio. And, the gram-negative *Barnesiella intestinihominis* was found to improve interferon-c–producing T cell infiltration in cancer lesions to enhance the antitumor effects of CP (Supplementary Table S1).

Together, these studies demonstrate that the response to ICB (PD1/PDL1) is regulated by gut microbiota (Figure 1). From these studies, we can conclude that at least three species (*Bifidobacterium*, *A. muciniphila*, *Faecalibacterium*) play an immune adjuvant role in the immunotherapy of PD-1. There may be more bacteria that play important roles in promoting or inhibiting the efficacy of checkpoint inhibitors, but these hypotheses need to be further examined. Overall, we can conclude that a healthy and diverse microbiota and the presence of some bacterial species contribute to the antitumor immune response. The efficacy of ICB was reduced when patients received antibiotic treatment before or soon after immune therapy. This finding provides new insight and ideas for the use of antibiotics in clinical treatment. In addition, it is obvious that not only single species but also the ecology and metabolism of the gut microbiota affect the response to immune therapy. It is possible that a new therapy targeting the microbiota could be developed to improve cancer treatment.

In terms of CTLA4, Vétizou et al. (2015) found that the microbiome experienced a rapid change when patients received anti-CTLA-4 and that the abundance of *Bacteroidales* and *Burkholderiales* decreased, while that of *Clostridiales* increased in the gut (Vétizou et al., 2015). Germ-free mice had a minor response to anti-CTLA-4 immunotherapy, but oral feeding of either *Bacteroides thetaiotaomicron* or *Bacteroides fragilis* to germ-free mice restored the therapeutic response to anti-CTLA4. Studies revealed that *B. thetaiotaomicron* and *B. fragilis* can trigger dendritic cell maturation and modulate IL-12–dependent TH 1 responses in the tumor-draining lymph nodes (Cramer and Bresalier, 2017). While the monoclonal antibody against CTLA-4 is effective, ipilimumab can cause subclinical colitis. Many factors contribute to such side effects, such as host homeostasis, immune response, and microbiota. The abundance of *Bacteroidetes* in new-onset, immune-mediated colitis patients who were administered anti-CTLA-4 therapy was significantly lower than in colitis-free individuals receiving ipilimumab (Dubin et al., 2016). The oral feeding of *B. fragilis* and *B. cepacian* to mice can restore the response to anti-CTLA4 and significantly decrease the extent of immune-mediated colitis. However, a single administration of either *B. fragilis* or *B. thetaiotaomicron* failed to produce the same effect. Moreover, Dubin et al. (2016) similarly revealed that the Bacteroidetes phylum plays a protective role against ipilimumab-associated colitis (Dubin et al., 2016). When germ-free mice receiving anti-CTLA-4 mAb were monopolized with *B. fragilis*, plasmacytoid DCs accumulated and matured in mesenteric lymph nodes, which promoted ICOS + Treg cells to proliferate in the lamina propria (Dasgupta et al., 2014; Vétizou et al., 2015). This may be a possible mechanism for the protective role of *B. fragilis* against ipilimumab-associated colitis.

Individual antibiotics play an important role in the immunotherapy of tumors and even affect the curative effects of immune agents by changing the composition of the gut microbiota. For example, when mice were administered vancomycin, the efficacy of CTLA-4 blockade was enhanced because vancomycin preserved the gram-negative *Burkholderiales* and *Bacteroidales* and decreased gram-positive bacteria in the gut (Vétizou et al., 2015). Zitvogel et al. (Pitt et al., 2017) explored the relationship between microbiota and the efficacy of anti-CTLA-4 treatment (Pitt et al., 2017). They found that the therapeutic efficacy of ipilimumab in germ-free mice largely depended on the gut microbiota, such as the activation of CD4+ T cells with treatment (Pitt et al., 2017). Ipilimumab could alter the composition of microbiota at the genus level in both patients and mice and the dominance of distinct *Bacteroides* spp., such as *B. fragilis*, was necessary for successful treatment of cancer (Pitt et al., 2017). The feces from patients who had received ipilimumab treatment led to the recovery of anti-CTLA-4 therapeutic efficacy in germ-free mice. The researchers found that *B. fragilis* did not induce the side effects of ipilimumab (Pitt et al., 2017). As a result of these observations, we can conclude that *B. fragilis* may be used to modulate the efficacy of anti-CTLA-4 therapy.

Synthetic CpG oligonucleotides (CpG-ON) are ligands for TLR9 on immune cells, which enhance the immune response to cancer cells and induce an antitumor effect. When patients were administered IL-10 receptor antibodies to prevent the immunosuppressive effects of tumor-infiltrating Treg cells, the effect of CpG-ON was potentiated (Guiducci et al., 2005; Stewart et al., 2013). CpG-ONs promote myeloid cells to secrete proinflammatory cytokines such as TNF and IL-12. TNF and IL-12 induce macrophage and dendritic cell infiltration to promote a proinflammatory state and cause rapid hemorrhagic necrosis. The body develops an antigen-specific adaptive T cell antitumor immunity to clear tumors in this proinflammatory microenvironment (Guiducci et al., 2005). In microbiota-depleted mice, CpG-ODNs and anti-IL-10R therapy for subcutaneous tumors are largely inefficient, and tumor-infiltrating myeloid cells cannot produce proinflammatory cytokines. Microbiota-depleted mice also have no efficient antitumor adaptive immunity and experience strong TNF-dependent hemorrhagic necrosis. However, the expression of genes encoding inflammatory factors and markers such as TNF and IL-12 was a major difference between conventionally raised mice and microbiota-depleted mice when CpG-ODNs were administered in tumor-infiltrating myeloid cell subsets. The frequencies of the gram-positive *Ruminococcus* and the gram-negative *Alistipes* genera favor TNF production. The frequencies of *Lactobacillus* sp., such as *Lactobacillus fermentum*, *Lactobacillus murinum*, and *Lactobacillus intestinalis* are negatively correlated with TNF production (Wallace et al., 2015). After mice were exposed to antibiotics, the recolonization of *Alistipes shahii* induced myeloid cells to produce TNF again in microbiota-depleted mice, but *L. fermentum* transplantation often impaired the TNF production of conventionally raised mice (Iida et al., 2013). These results indicate that different bacterial species can have opposite effects, although completely eliminating the gut microbiota abolishes the ‘training’ of myeloid cells to respond to CpG-ODNs. Thus, probiotics could help modulate the response to immunotherapies by changing the frequencies of individual species.

### Chemotherapy

Not unexpectedly, the microbial composition of patients can be altered by chemotherapy, but it is unclear whether the altered microbiome affects a patients’ prognosis. According to previous studies, the efficacy of various conventional chemotherapeutics can be influenced by some specific microbiota. Currently, the goals of the pharmaceutical and biotechnology industries are to improve the efficiency, and reduce the toxicity, of chemotherapy and immunotherapy in clinical practice. In the near future, microbial drug targets have the potential to ease the adverse effects of chemotherapy drugs on the GI tract.

The tumor-retardation effects of oxaliplatin (platinum chemotherapeutic) depend on microbiota. Oxaliplatin efficacy was attenuated due to reduced intratumoral ROS generation in germ-free mice (Iida et al., 2013). Moreover, when people were treated with antibiotics, the recruitment of immune cells that are important for mediating tumor regression was decreased, and their proinflammatory potential also decreased. This finding suggests that the microbiota mediated immunomodulatory effects in response to chemotherapeutic compounds.

Cyclophosphamide (CP) is an alkylating agent commonly used for chemotherapy. CP induces commensals to translocate into secondary lymphoid organs due to the disruption of the intestinal barrier and the decrease of small intestinal villus height. Viaud et al. (2013) found that the antitumor efficacy of CP was attenuated in germ-free mice or antibiotic-treated mice (Viaud et al., 2013). The antibiotics selectively working on gram-positive bacteria significantly reduced CP efficacy compared with antibiotics targeting gram-negative bacteria. Thus, specific gram-positive bacteria (*E. hirae*, *Lactobacillus johnsonii*, *L. murinus*, and segmented filamentous bacteria) were identified as essential to regulate the antitumor efficacy of CP in a non-metastasizing sarcoma mouse model. *E. hirae* translocation has been shown to improve the intra-tumoral CD8/Treg ratio (Iida et al., 2013). At the same time, the gram-negative *Barnesiella intestinihominis* was found to improve interferon-c–producing T cell infiltration in cancer lesions to enhance the antitumor effects of CP (Daillère et al., 2016). Interestingly, when patients with advanced ovarian and lung cancer have a specific TH 1 cell memory response to *B. intestinihominis* and *E. hirae*, they are predicted to have longer progression-free survival. Importantly, more studies should be conducted to find an optimized microbiota cocktail including *Enterococcus* and *Barnesiella* coadministered with CP and other alkylating agents. In the near future, these bacterial compounds or their specific products/metabolites that modulate the immune response may be developed to improve chemotherapeutic efficacy.

## The Role of Gut Microbiota in the Toxicity of Cancer Therapy (Including Chemotherapy, Radiotherapy, and Immunotherapy)

### Chemotherapy

Some side effects resulting from chemotherapeutic compounds are so serious that patients cannot receive a sufficient dose of compounds or a sufficient duration of treatment. Irinotecan (topoisomerase I inhibitor) hinders DNA replication, particularly in rapidly dividing cells, and is administered to treat pancreatic cancer and CRC. The metabolic process of irinotecan *in vivo* is as follows: (1) irinotecan is metabolized from a prodrug into the active working chemotherapeutic agent SN38; (2) the liver glucuronidates SN38 into the inactive form SN38-G and excretes it into the GI tract. In the human gut, microbiota can reactivate SN38 by secreting b-glucuronidase enzymes that hydrolyze the glucuronic acid moiety in the GI lumen. Increased SN38 levels cause serious diarrhea, and patients often need to de-escalate and frequently adjust doses. *Clostridium* species decrease from the initial time of irinotecan therapy to recovery on day 7, but the abundance of *Bifidobacterium* and *Lactobacillus* species is persistently reduced (Lin et al., 2012). Interestingly, germ-free mice can receive more doses of irinotecan and exhibit less GI damage than conventional mice with intact microbiota (Brandi et al., 2006). Small-molecule inhibitors, which are innocuous to either human cells or bacteria, inhibit bacterial b-glucuronidases and do not cross-react with human b-glucuronidases (Wallace et al., 2010, 2015; Roberts et al., 2013). Preclinical studies revealed that mice concurrently administered irinotecan and b-glucuronidase inhibitors were free from irinotecan-induced diarrhea (Wallace et al., 2010). These findings indicate that the side effects of multiple chemotherapeutics may diminish with gut microbiota.

The relationship between intestinal dysbiosis and specific chemotherapeutic agents has been explored in animal models, and 5-fluorouracil (5-FU) is one of the best studied agents in colorectal cancer therapies. 5-FU interferes with the synthesis of thymidylate and inhibits DNA synthesis during DNA replication and repair (Longley et al., 2003). Studies have shown that mice receiving 5-FU chemotherapy exhibit dysbiosis. Specifically, the abundance of *Staphylococcus* and *Clostridium* species increased and that of *Bacteroides* and *Lactobacillus* decreased after administration with 5-FU (Stringer et al., 2009b). Multiple animal studies showed that the abundance of Enterobacteriaceae (facultative gram-negative bacteria) increased after either irinotecan or 5-FU therapy (Stringer et al., 2009a; Takemura et al., 2014). However, these studies relied on targeted PCR or culture methods and cannot evaluate the influence of chemotherapy on the extensive gut microbiota. It is still a challenge to manipulate probiotics to treat intestinal dysbiosis in 5-FU therapy.

Severe side effects induced by doxorubicin, such as intestinal mucositis and cardiomyopathy, are related to significant changes in the microbiota of the oral cavity and the intestinal tract (Napeñas et al., 2010; Rigby et al., 2016). Studies have revealed that bacterial muramyl dipeptide prevented doxorubicin-induced mucosal damage by stimulating NOD2 (Nigro et al., 2014). Clinical practice shows that adipose tissue and fat metabolism are influenced in many tumor patients, resulting in cachexia (Das et al., 2011; de Matos-Neto et al., 2015). Pancreatic beta-cell mass, uptake of lipids, and adipose tissue inflammation are regulated by the gut microbiota. Cancer therapy can exacerbate the serious effects of cancer-induced cachexia (Antoun et al., 2010; Toledo et al., 2016), but some chemotherapeutic agents can also directly cause muscle wasting and multi-organ failure that resemble cancer-induced cachexia (Garcia et al., 2008; Toledo et al., 2016). We do not completely understand the cachexia mechanism underlying these conditions, but this observation raises the possibility that the close relationship between energy metabolism and gut microbiota could be a therapeutic target, as the microbiota composition could affect the pathogenesis of this condition (Bindels and Delzenne, 2013; Klein et al., 2013; de Matos-Neto et al., 2015). Probiotics can improve body weight in mice and patients with cancer-associated cachexia (Yeh et al., 2013; Varian et al., 2016). Recent studies in mice have found that colonization by the *E. coli* strain O21:H + in the gut protects against muscle wasting induced by intestinal damage (Schieber et al., 2015). Modulation between gut microbiota and homeostasis could be an effective clinical means to treat cancer-associated diseases, such as cachexia and anorexia, and adverse cancer treatment effects. Additional mechanism studies and rigorous clinical trials are necessary.

### Radiotherapy

Ionizing radiation therapy (RTX) is an effective way to treat tumors based on its genotoxic effect on tumor cells. Immunogenic tumor cell death can be induced by local irradiation, and systemic immunity and inflammation are also promoted (Demaria and Formenti, 2012; Kroemer et al., 2013). Unfortunately, ionizing radiation also induces some side effects, including genomic instability, bystander effects on nearby cells, and systemic radio-associated immune and inflammatory reactivity (Azzam and Little, 2004). Although there has been considerable progress in the development of ionizing radiation therapy, the main limitations are the safety and effectiveness of RTX and heterogeneity in the therapeutic sensitivity of diverse cancer types and kinds of side effects with RTX (Deng et al., 2014; Baird et al., 2016).

Healthy tissues are also damaged by RTX, which is more obvious in actively proliferating tissues (Barker et al., 2015). RTX alters the microbiota composition, breaks the intestinal barrier, and causes apoptosis in intestinal crypts (Barker et al., 2015). The pathogenesis of oral mucositis, enteritis, colitis, diarrhea, and bone marrow failure in patients and mice receiving RTX is associated with alterations in the epithelial surface microbiota composition (Touchefeu et al., 2014; Ó Broin et al., 2015). The serious oral mucositis and enteropathy induced by RTX may limit therapy completion. Some studies have shown that irradiation-mediated intestinal toxicity is regulated by TLR3 in dsRNA. TLR3 mice receiving ionizing radiation survived longer and suffered less severe intestinal toxicity compared with wild type mice, suggesting that suppression of TLR3 signaling may decrease the gastrointestinal damage induced by radiation (Adams, 2009; Takemura et al., 2014). In contrast, TLR2-activating microorganisms in mice, such as the probiotic *Lactobacillus rhamnosus* GG (Ciorba et al., 2012), have been shown to protect the intestinal mucosa against radiotherapy-induced toxicity by driving cyclooxygenase 2-expressing cells from the intestinal villi to the bottom of the intestinal crypts and producing ROS to activate the cytoprotective NRF2 system (Jones et al., 2013, 2015). In some clinical studies, probiotics have been shown to help prevent radiation-related enteropathy. Preparations containing *B. bifidum*, *L. acidophilus*, *Lactobacillus casei*, and the VSL#3 formulation containing *Streptococcus*, *Lactobacillus*, and *Bifidobacterium* spp. have been proven to reduce radiation-induced gut toxicity, such as diarrhea (Delia et al., 2007; Touchefeu et al., 2014). Head and neck cancer patients who were administered radiation and chemotherapy treatment and received *Lactobacillus brevis* oral-treatments with CD2 lozenges had a lower incidence of mucositis and greater treatment completion. All of these findings raise the possibility that probiotics could become an adjuvant therapy for cancer treatment.

Studies have shown that intestinal microbiota have a significant effect on total body irradiation (Crawford and Gordon, 2005). Irradiation drives fewer endothelial cells of the intestinal mucosa into apoptosis and induces less lymphocyte infiltration in germ-free mice than in conventional mice (Crawford and Gordon, 2005). This finding indicates that gut commensals can play a negative role in resistance to the enteric toxicity of TBI in germ-free mice. However, the production of angiopoietin-like 4 (ANGPTL4), a protein inhibitor of lipoprotein lipase, is one of the major mechanisms resulting in the resistance of germ-free mice to TBI. The expression of ANGPTL4 is restrained by the gut microbiota in conventional mice (Crawford and Gordon, 2005). The transcription of Angptl4 is administered by the PPAR family in response to small chain fatty acid-producing bacteria (Grootaert et al., 2011; Korecka et al., 2013). Further exploration revealed that probiotic bacteria that induce Angptl4 expression include *Streptococcus*, *Lactobacillus*, and *Bifidobacterium* spp. and these render both germ-free mice and conventional mice resistant to radiotherapy toxicity.

We can conclude that gut microbiota regulates the response and repair of irradiation-induced damage. Future research will be invaluable to inform the alleviation of radiotherapy-collateral toxicity, the increase of therapeutic effectiveness to better understand the regulation mechanisms, and the therapeutic manipulation of commensal microbiota.

## Application to Clinical Practice

Several clinical trials are ongoing. The “Intestinal Microflora in Lung Cancer After Chemotherapy” trial was launched by Shandong University to explore how probiotics modulate the gut microflora and immune status in lung cancer patients who need chemotherapy (ClinicalTrials.gov, 2018). Concurrently, the University of Arkansas carried out a project named “Gut Microbiome and Gastrointestinal Toxicities as Determinants of Response to Neoadjuvant Chemo for Advanced Breast Cancer” (ClinicalTrials.gov, 2018). The goal of this research was to study whether normal gut bacteria help the body fight cancer. S&D Pharma Ltd., will conduct a project titled “Prevention of Febrile Neutropenia by Synbiotics in Pediatric Cancer Patients (FENSY)” to find new options for increasing the quality of healthcare for pediatric cancer patients (ClinicalTrials.gov, 2018). Febrile neutropenia (FN) is a major treatment-related complication and a life-threatening condition for cancer patients receiving intensive chemotherapy. One of the main sources of infection during neutropenia is the endogenous flora. According to existing human and animal studies, probiotics probably not only decrease the degree of enrichment of the pathogenic bacteria colonizing the gut but may also reduce the duration of neutropenia. Although a significant number of studies have shown that probiotic treatment is effective, evidence of the safety of probiotics is still insufficient, especially in immunocompromised patients. This new study will explore the safety and practicability of probiotics in cancer treatment (ClinicalTrials.gov, 2018).

## Perspective

In general, abundant gut microbiota play a regulatory role in tumor therapy, including enhancing the sensitivity of patients to immunotherapy, reducing side effects of chemotherapeutic agents, and lightening radiation injuries. However, the effects of other mucosal barrier microbes on the body are still not clear. Many existing studies have revealed mechanisms of the gut microbiota that affect carcinogenesis, inflammation, immunity, and therapy response at the local level. However, it is still not known how microbiota colonizing distant epithelial barriers regulate not only carcinogenesis and immunity but also the physiological functions of many organs. Most studies investigating how microbiota modulate cancer therapy have been carried out in mice, and how to translate these academic findings to the clinic is still a challenge. The change in the monogenus does not explain the mechanisms behind the body’s corresponding changes. The entire body is affected by the gut microbiota. Although mice transplanted with human microbiota have pathological and immune responses similar to humans, they are not identical to those in humans (Smith et al., 2007). For example, *Bifidobacterium* activates immune cells through two different functional innate immune receptors, TLR2 and TLR9, in the mouse, but the cellular expression of TLR9 is very different between mice and humans. TLR9 is expressed on plasmacytoid dendritic cells and B cells in humans, whereas it is expressed in all myeloid and dendritic cells in mice (Kadowaki et al., 2001). Thus, while activation of TLR9 by *Bifidobacterium spp.* in mice has immunostimulating activity, we cannot assume the same is true in humans. Once the most beneficial microbiota compositions in various clinical conditions have been identified, it may be possible to use microbiota composition as a biomarker, a diagnostic tool, or a therapeutic target. Targeted interventions in the microbiome using probiotics may be used for cancer prevention in particularly high-risk populations. Several clinical trials are ongoing. The ultimate goal is to develop a microbe therapy that both promotes anticancer therapy and reduces systemic toxicity. Thus, therapeutic intervention targeting the microbiota will be one of the next frontiers for precise and personalized therapies for cancer treatment.

## Author Contributions

FJ and CY contributed to the conception of the study and drafted the work for the manuscript framework, and agreed to publish the manuscript and be accountable for all aspects of the work in ensuring that questions related to the accuracy or integrity of any part of the work are appropriately investigated and resolved. GD contributed significantly to manuscript preparation. WM performed the data analyses and wrote the manuscript. QM and WX helped to perform the analysis with constructive discussions. All authors listed have made a substantial, direct and intellectual contribution to the work, and approved it for publication.

## Conflict of Interest Statement

The authors declare that the research was conducted in the absence of any commercial or financial relationships that could be construed as a potential conflict of interest.
