# Supplementary material for: Gut Microbiota Shapes the Efficiency of Cancer Therapy
Source: Front Microbiol. 2019 Jun 25;10:1050. doi: 10.3389/fmicb.2019.01050 (PMC6604670; doi:10.3389/fmicb.2019.01050)
Supplement: Supplementary file 1 [file Table_1.docx]

**Supplementary Table 1.** Changes of the immune system of tumor by gut microbiota

| Therapy | gut microbiota |  | Changes of the immune system |
| --- | --- | --- | --- |
| Anti-PD-L1 | *Akkermansia muciniphila*  *Faecalibacterium*  *Enterococcus hirae*  *Bifidobacterium* | | The microbiota enhance anti-PD-L1 efficacy by reactivating dendritic cells. Dendritic cells can boost CD8-positive T cell responses and increase the number of gut-tropic CD4 + T cells to defeat tumors(Sivan et al., 2015; Routy et al., 2018). |
| Anti-CTLA4 | *Bacteroides fragilis*  *Burkholderia cepacia*  *Bacteroides thetaiotaomicron* | | *Bacteroides thetaiotaomicron or Bacteroides fragilis restored the therapeutic response to anti-CTLA4 in germ-free mice* (Vétizou et al., 2015).  Studies revealed that *B. thetaiotaomicron* and *B. fragilis* can trigger dendritic cell maturation and modulate IL-12–dependent Th 1 responses in the tumor-draining lymph nodes (Cramer and Bresalier, 2017).  The microbiota are associated with side effects of immunotherapy. *Bacteroidetes* can significantly decrease the extent of immune-mediated colitis (Dubin et al., 2016). *Bacteroides fragilis* accumulate and mature plasmacytoid DCs that can promote ICOS + Treg cells to proliferate in the lamina propria. |
| Cyclophosphamide | *Enterococcus.hirae, Lactobacillus johnsonii,*  *Lactobacillus murinus* and segmented filamentous bacteria | | *E. hirae* translocation could improve the intratumoral CD8/ Treg ratio (Iida et al., 2013). The Gram-negative bacterium *Barnesiella intestinihominis* was found to improve interferon-c–producing T cell infiltration in cancer lesions to enhance the antitumor effects of CP (Daillère et al., 2016). |

**REFERENCES**

Cramer, P., and Bresalier, R. S. (2017). Gastrointestinal and Hepatic Complications of Immune Checkpoint Inhibitors. *Curr Gastroenterol Rep* 19, 3. doi: 10.1007/s11894-017-0540-6

Daillère, R., Vétizou, M., Waldschmitt, N., Yamazaki, T., Isnard, C., Poirier-Colame, V., et al. (2016). Enterococcus hirae and Barnesiella intestinihominis Facilitate Cyclophosphamide-Induced Therapeutic Immunomodulatory Effects. *Immunity* 45, 931-943. doi: 10.1016/j.immuni.2016.09.009

Dubin, K., Callahan, M. K., Ren, B., Khanin, R., Viale, A., Ling, L., et al. (2016). Intestinal microbiome analyses identify melanoma patients at risk for checkpoint-blockade-induced colitis. *Nat Commun* 7, 10391. doi: 10.1038/ncomms10391

Iida, N., Dzutsev, A., Stewart, C. A., Smith, L., Bouladoux, N., Weingarten, R. A., et al. (2013). Commensal bacteria control cancer response to therapy by modulating the tumor microenvironment. *Science* 342, 967-970. doi: 10.1126/science.1240527

Routy, B., Le, C. E., Derosa, L., CPM, D., Alou, M. T., Daillère, R., et al. (2018). Gut microbiome influences efficacy of PD-1-based immunotherapy against epithelial tumors. *Science* 359, 91-97. doi: 10.1126/science.aan3706

Sivan, A., Corrales, L., Hubert, N., Williams, J. B., Aquino-Michaels, K., Earley, Z. M., et al. (2015). Commensal Bifidobacterium promotes antitumor immunity and facilitates anti-PD-L1 efficacy. *Science* 350, 1084-1089. doi: 10.1126/science.aac4255

Vétizou, M., Pitt, J. M., Daillère, R., Lepage, P., Waldschmitt, N., Flament, C., et al. (2015). Anticancer immunotherapy by CTLA-4 blockade relies on the gut microbiota. *Science* 350, 1079-1084. doi: 10.1126/science.aad1329
